# THE INTERSECTION OF OLDER ADULTS, DISABILITIES, AND MENTAL HEALTH

**DOI:** 10.1093/geroni/igae098.3901

**Published:** 2024-12-31

**Authors:** Onyinye Mbanefo, Pamela Teaster

**Affiliations:** Virginia Tech, Blacksburg, Virginia, United States; Virginia Tech., BLACKSBURG, Virginia, United States

## Abstract

The number of individuals aged 65 and older is projected to reach 82 million by 2050. This demographic shift is accompanied by an increase in the prevalence of disabilities and mental health issues among older adults. Nearly 46% of adults aged 75 and older report having a disability, and 25% of those aged 65 and older are living with a mental health condition such as anxiety or depression. These challenges are exacerbated by cumulative disadvantage, ageism, and a shortage of mental health providers trained to work with this population. To address these issues, we apply a strengths-based approach to explore the intersection of aging, disability, and mental health. Our approach emphasizes the resilience and potential of older adults, challenging the dominant narratives of decline and loss. Utilizing the Socio-Ecological Model, we examine how individual, relational, community, and societal factors influence the mental health of older adults, highlighting the need for integrated and holistic care strategies. We explore key concepts such as cumulative advantage and disadvantage, ageism, and intersectionality, which are critical in understanding the complexities faced by older adults. Our work advocates for comprehensive mental health care that addresses both the physical and psychological well-being of older adults, promoting dignity, equity, and quality of life. Our findings suggest that a strengths-based approach can effectively enhance mental health outcomes for older adults, providing a framework for mental health practitioners and policymakers alike.

